# Comparative genomic analysis of *Klebsiella pneumoniae* subsp. *pneumoniae* KP617 and PittNDM01, NUHL24835, and ATCC BAA-2146 reveals unique evolutionary history of this strain

**DOI:** 10.1186/s13099-016-0117-1

**Published:** 2016-07-11

**Authors:** Taesoo Kwon, Young-Hee Jung, Sanghyun Lee, Mi-ran Yun, Won Kim, Dae-Won Kim

**Affiliations:** School of Biological Sciences, Seoul National University, 1 Gwanak-ro, Gwanak-gu, Seoul, 151-742 Republic of Korea; Division of Antimicrobial Resistance, Korea National Institute of Health, Cheongju, 363-951 Republic of Korea; Division of Biosafety Evaluation and Control, Korea National Institute of Health, Cheongju, 363-951 Republic of Korea

**Keywords:** *Klebsiella pneumoniae*, OXA-232, NDM-1, Carbapenemases

## Abstract

**Background:**

*Klebsiella pneumoniae* subsp. *pneumoniae* KP617 is a pathogenic strain that coproduces OXA-232 and NDM-1 carbapenemases. We sequenced the genome of KP617, which was isolated from the wound of a Korean burn patient, and performed a comparative genomic analysis with three additional strains: PittNDM01, NUHL24835 and ATCC BAA-2146.

**Results:**

The complete genome of KP617 was obtained via multi-platform whole-genome sequencing. Phylogenetic analysis along with whole genome and multi-locus sequence typing of genes of the *Klebsiella pneumoniae* species showed that KP617 belongs to the WGLW2 group, which includes PittNDM01 and NUHL24835. Comparison of annotated genes showed that KP617 shares 98.3 % of its genes with PittNDM01. Nineteen antibiotic resistance genes were identified in the KP617 genome: *bla*_*OXA*-*1*_ and *bla*_*SHV*-*28*_ in the chromosome, *bla*_*NDM*-*1*_ in plasmid 1, and *bla*_*OXA*-*232*_ in plasmid 2 conferred resistance to beta-lactams; however, colistin- and tetracycline-resistance genes were not found. We identified 117 virulence factors in the KP617 genome, and discovered that the genes encoding these factors were also harbored by the reference strains; eight genes were lipopolysaccharide-related and four were capsular polysaccharide-related. A comparative analysis of phage-associated regions indicated that two phage regions are specific to the KP617 genome and that prophages did not act as a vehicle for transfer of antimicrobial resistance genes in this strain.

**Conclusions:**

Whole-genome sequencing and bioinformatics analysis revealed similarity in the genome sequences and content, and differences in phage-related genes, plasmids and antimicrobial resistance genes between KP617 and the references. In order to elucidate the precise role of these factors in the pathogenicity of KP617, further studies are required.

**Electronic supplementary material:**

The online version of this article (doi:10.1186/s13099-016-0117-1) contains supplementary material, which is available to authorized users.

## Background

*Klebsiella pneumoniae* is a Gram-negative, non-motile, encapsulated, facultative anaerobic bacterium, which belongs to the family Enterobacteriaceae. *K. pneumoniae* is found in the normal flora of the mouth, skin, and intestines; however, this bacterium may act as an opportunistic pathogen, causing severe nosocomial infections such as septicemia, pneumonia, and urinary tract infections in hospitalized and immune-comprised patients with chronic ailments [1, 2].

Beta-lactam antibiotics, used as therapeutic agents against a broad range of bacteria, bind to the penicillin-binding protein and inhibit biosynthesis of the bacterial cell membrane. However, the extended spectrum β-lactamases (ESBLs) and carbapenemases confer resistance to penicillin, cephalosporins, or carbapenem [3, 4]. The β-lactamases are divided into four classes on the basis of the Ambler scheme: class A (*Klebsiella pneumoniae* carbapenemase, KPC; imipenem-hydrolyzing β-lactamase, IMI; *Serratia marcescens* enzyme, SME; *Serratia fonticola* carbapenemase, SFC), class B (Verona integron-encoded metallo-β-lactamase, VIM; imipenem-resistant *Pseudomonas*, IMP; New Delhi metallo-β-lactamase, NDM), class C (AmpC-type β-lactamase, ACT; cephamycin-hydrolyzing β-lactamase, CMY), and class D (oxacillinase, OXA) [5] are composed of transposon, cassettes, and integrons and transferred within and between species by HGT (horizontal gene transfer). Numerous carbapenemase-producing bacteria similarly harbor drug resistance genes that are transferred to other strains by horizontal gene transfer [6, 7]; infections caused by such multi-drug-resistant bacteria are difficult to treat [8]. The emergence of the novel carbapenemase NDM-1 (the New Delhi metallo-β-lactamase) is of great concern, as no therapeutic agents are available to treat infections caused by NDM-1-producing bacterial strains [9]. NDM-1-producing *K. pneumoniae* strains were first isolated from a Swedish patient who had travelled to India in 2009 [10]. Since then, NDM-1 has been reported to be produced by various species of Enterobacteriaceae, such as *K. pneumoniae, Escherichia coli, Enterobacter* spp. and *Acinetobacter* spp., in numerous countries [11].

The carbapenem-hydrolyzing β-lactamase OXA-232, which was first reported in *E. coli* and two *K. pneumoniae* strains [12], belongs to the OXA-48-like family. Carbapenemase-producing Gram-negative bacteria are often multi-drug resistant [13]. *K. pneumoniae* isolates that coproduce OXA-48-like β-lactamase and NDM-1 have been isolated in numerous countries [14–16]. Recently, *K. pneumoniae* isolates coproducing two carbapenemases, *bla*_*NDM*-*1*_ and *bla*_*OXA*-*232*_, have been identified in several countries; of these, two isolates originating in India were recovered in the USA and Korea, in January 2013, and sequenced [16, 17] but not studied yet the characteristics in the context of genomic contents by comparing these isolates. In the present study, we performed a comparative analysis of the genomes of these isolates.

## Methods

### Isolation and serotyping of strains

In January 2013, a 32-year-old man was hospitalized in the Intensive Care Unit of a general hospital in Seoul, Korea, two days after suffering burns during a visit to India. *K. pneumoniae* was isolated from his wound and another patient in the same room became infected with the same strain [18]. The *K. pneumoniae* isolate was identified as the KP617 strain belonging to the sequence type (ST)14, and found to coproduce NDM-1 and OXA-232, which conferred resistance to ertapenem, doripenem, imipenem, and meropenem (MICs: >32 mg/L). The *K. pneumoniae* strains PittNDM01 [17], NUHL24835 [19], and ATCC BAA-2146 [20] were used as reference strains for comparative genomic analysis.

### Library preparation and whole-genome sequencing

Whole-genome sequencing of KP617 was performed using three platforms: Illumina-HiSeq 2500, PacBio RS II, and Sanger sequencing (GnC Bio: Daejeon, Republic of Korea) [16]. Sanger sequencing was used for the construction of a physical map of the genome.

### Genome assembly and annotation

A hybrid assembly was performed using the Celera Assembler (version 8.2) [21] and a fosmid paired-end sequencing map was used to confirm the assembly. The final assembly was revised using proovread (version 2.12) [22]. An initial annotation of the KP617 genome was generated using the RAST (Rapid Annotation using Subsystem Technology, version 4.0) server pipeline [23]. The genomes of three *K. pneumoniae* strains, PittNDM01, NUHL24835, and ATCC BAA-2146, were annotated using the RAST server pipeline. In order to compare the total coding sequences (CDSs) of KP617 with those of the three *K. pneumoniae* strains, the sequence-based comparison functionality of the RAST server was utilized.

### Phylogenetic analysis

Concatenated whole genomes of 44 *K. pneumoniae* strains, including KP617, and multi-locus sequence typing (MLST) of seven genes [24, 25] were used for the calculation of evolutionary distances. The seven genes used for MLST were as follows: *gapA*, *infB*, *mdh*, *pgi*, *phoE*, *rpoB* and *tonB*. Multiple sequence alignments were performed using Mugsy (version 1.2.3) [26]. The generalized time-reversible model [27] + CAT model [28] (FastTree Version 2.1.7) [29] was used to construct approximate maximum-likelihood phylogenetic trees. The resulting trees were visualized using FigTree (version 1.3.1) (http://tree.bio.ed.ac.uk/software/figtree/).

### Comparison of genomic structure

The chromosome and plasmids of KP617 and the reference strains were compared using Easyfig (version 2.2.2) [30]. Whole-genome nucleotide alignments were generated using BLASTN to identify syntenic genes. The syntenic genes and genomic structures were visualized using Easyfig. A stand-alone BLAST algorithm was used to analyze the structure of the genes of interest, i.e. the OXA-232- and NDM-1 carbapenemase-encoding genes.

### Identification of the antimicrobial resistance genes

We identified the antibiotic resistance genes using complete sequences of chromosomes and plasmids of four *K. pneumoniae* isolates: KP617, PittNDM01, NUHL24853 and ATCC BAA-2146 using ResFinder 2.1 (https://cge.cbs.dtu.dk/services/ResFinder/) [31].

### Analysis of virulence factors and phage-associated regions

The virulence factor-encoding genes were searched against the virulence factor database (VFDB) [32] using BLAST with an e-value threshold of 1e-5. Homologous virulence factor genes with a BLAST Score Ratio (BSR) of ≥0.4 were selected. The BSR score was calculated using our in-house scripts. Phage-associated regions in the genome sequences of the four *K. pneumoniae* strains were predicted using the PHAST server [33]. Three scenarios for the completeness of the predicted phage-associated regions were defined according to how many genes/proteins of a known phage the region contained: intact (≥90 %), questionable (90–60 %), and incomplete (≤60 %).

### Quality assurance

Genomic DNA was purified from a pure culture of a single bacterial isolate of KP617. Potential contamination of the genomic library by other microorganisms was assessed using a BLAST search against the non-redundant database.

## Results and discussion

### General features

**Fig. 1:**
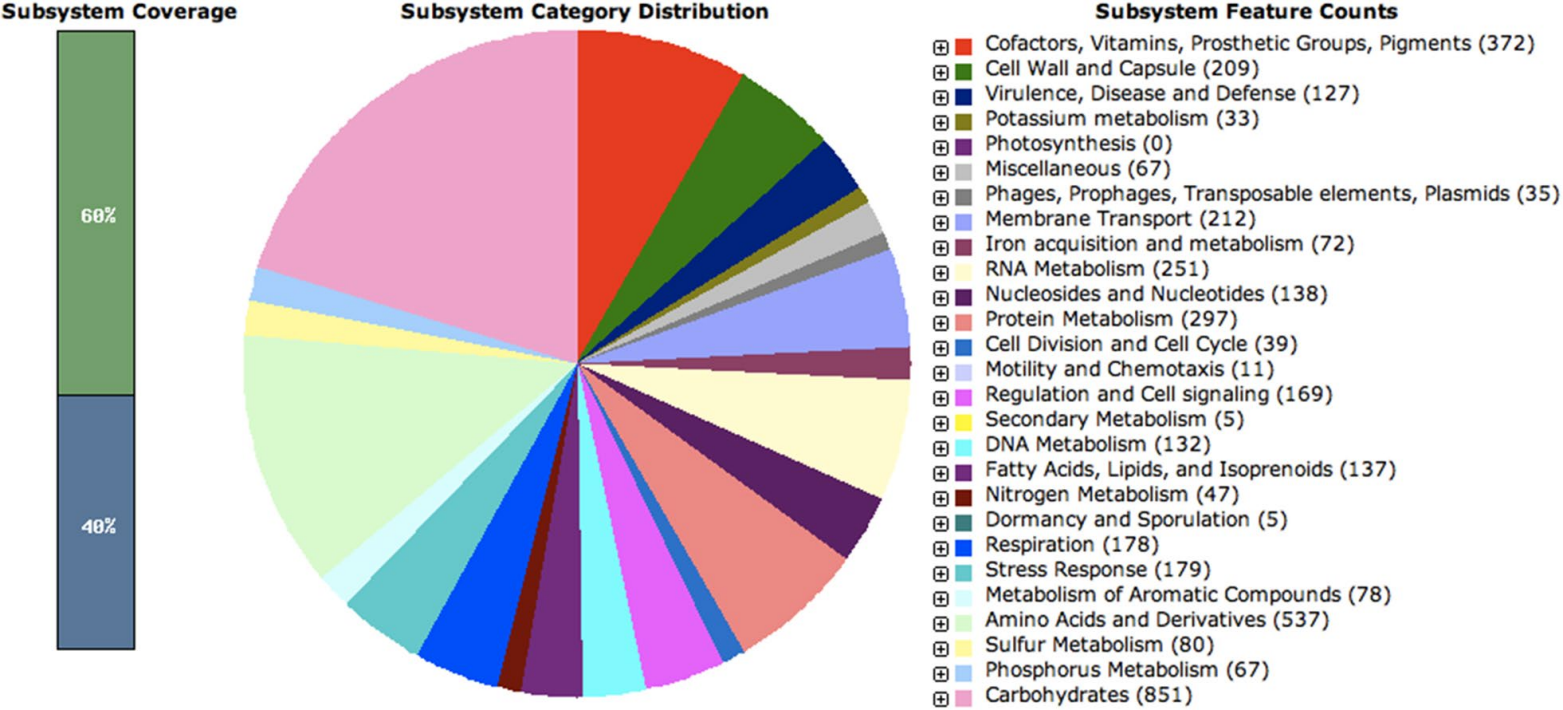
Subsystem category distribution of KP617 based on SEED databases

**Fig. 2:**
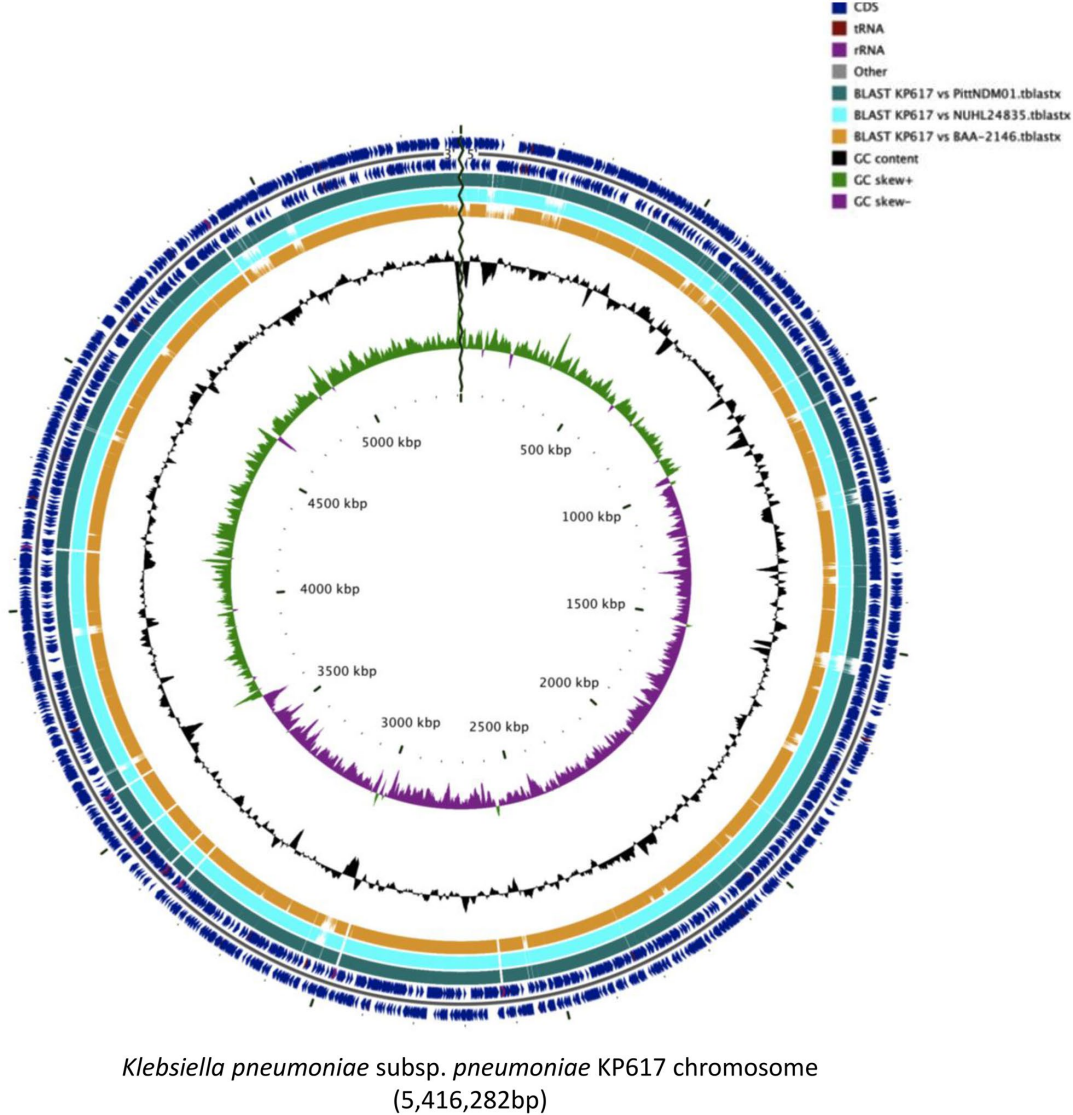
Circular map of the KP617 chromosome. Circular map of the KP617 genome, generated using cgview (version 2.2.2); from outside to inside, the tracks display the following information: CDSs of KP617 on the + strand (1); CDSs of KP617 on the − strand (2); tblastx result against PittNDM01 (3), tblastx result against NUHL24835 (4), tblastx result against ATCC BAA-2146 (5), GC content (6), GC skew with + value (*green*) and − value (*purple*) (7)

A total of 316,881,346 (32,005,015,946 bp) paired-end reads were generated using Illumina-HiSeq 2500. Using the PacBio RS II platform, 46,134 (421,257,386 bp) raw reads were produced. The complete genome of KP617 consists of a 5,416,282-bp circular chromosome and two plasmids of 273,628 bp and 6141 bp in size. The genomic features of KP617 and the reference strains are summarized in Table 1. Based on a RAST analysis, 5024 putative open reading frames (ORFs) and 110 RNA genes on the circular chromosome (Figs. 1, 2; Additional file 1: Table S1), 342 putative ORFs on plasmid 1, and 9 putative ORFs on plasmid 2 were identified.

**Table 1:** Genomic features of *Klebsiella pneumoniae* KP617 and other strains

Strain	KP617	PittNDM01	NUHL24835	ATCC BAA-2146
Genome (Mb)	5.69	5.81	5.53	5.78
% GC (chromosome)	57.4	57.5	57.4	57.3
Total open reading frames	5375	4940	5191	5883
Plasmids	2	4	2	4

**Fig. 3:**
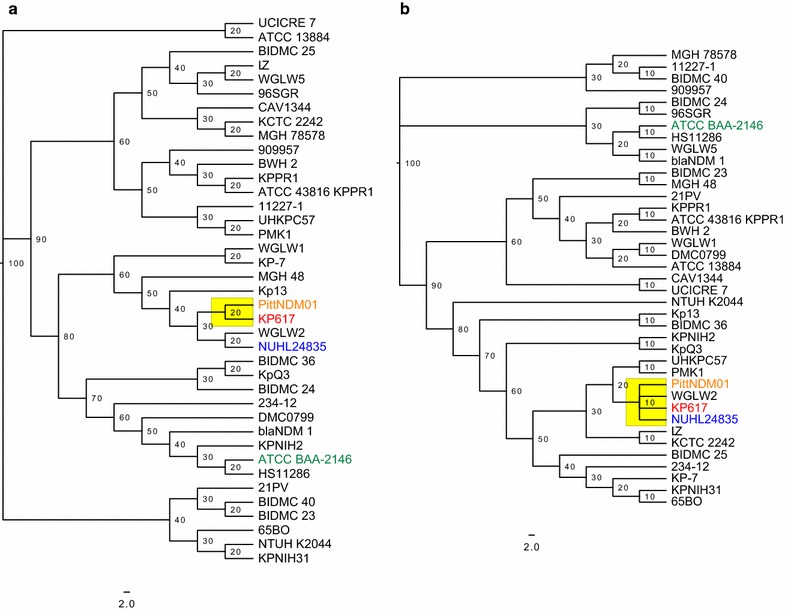
Phylogenetic tree of *Klebsiella pneumoniae*, **a** whole-genome phylogenetic tree; **b** MLSA phylogenetic tree; the scale represents the number of substitutions per site

Comparison of KP617 and the reference strains based on sequence similarity (percent identity ≤80) showed that 32 genes are unique for KP617, and that most of the functional genes of this strain are also conserved in the reference strains. The genes unique to the KP617 strain, such as the SOS-response repressor and protease LexA (EC 3.4.21.88), integrase, and phage-related protein were identified as belonging to the genome of the prophage Salmonella phage SEN4 (GenBank accession: NC_029015). When the KP617 genome was compared with that of the PittNDM01 strain, which represents the closest neighbor of the former strain on the phylogenetic tree (Figs. 3a, b), 94 genes showed a percent similarity of below 80; most of these were phage protein-encoding genes. These results indicate that the presence of prophage DNA is an important feature of the KP617 genome.

### Phylogenetic analysis

The whole-genome phylogenetic analysis indicated that KP617 is evolutionarily close to PittNDM01 and NUHL24835, and that the strains belong to the WGLW2 group. However, KP617 was found to be evolutionarily distant from ATCC BAA-2146 (Fig. 3). Concordantly, MLST-based phylogenetic analysis revealed that while KP617, PittNDM01, and NUHL24835 belong to the same group [sequence type (ST)14], ATCC BAA-2146 belongs to the HS11286 group, ST 11 [20]. The only difference between the whole-genome phylogenetic tree and the MLST-based phylogenetic tree was the divergence time within the same group; MLST-based phylogeny did not reveal the minor details of genomic evolution such as the divergence between KP617, PittNDM01 and NUHL24835 in the whole-genome phylogeny. The difference was attributed to horizontal gene transfer in regions not covered by the MLST genes.

### Comparison of genome structures

**Fig. 4:**
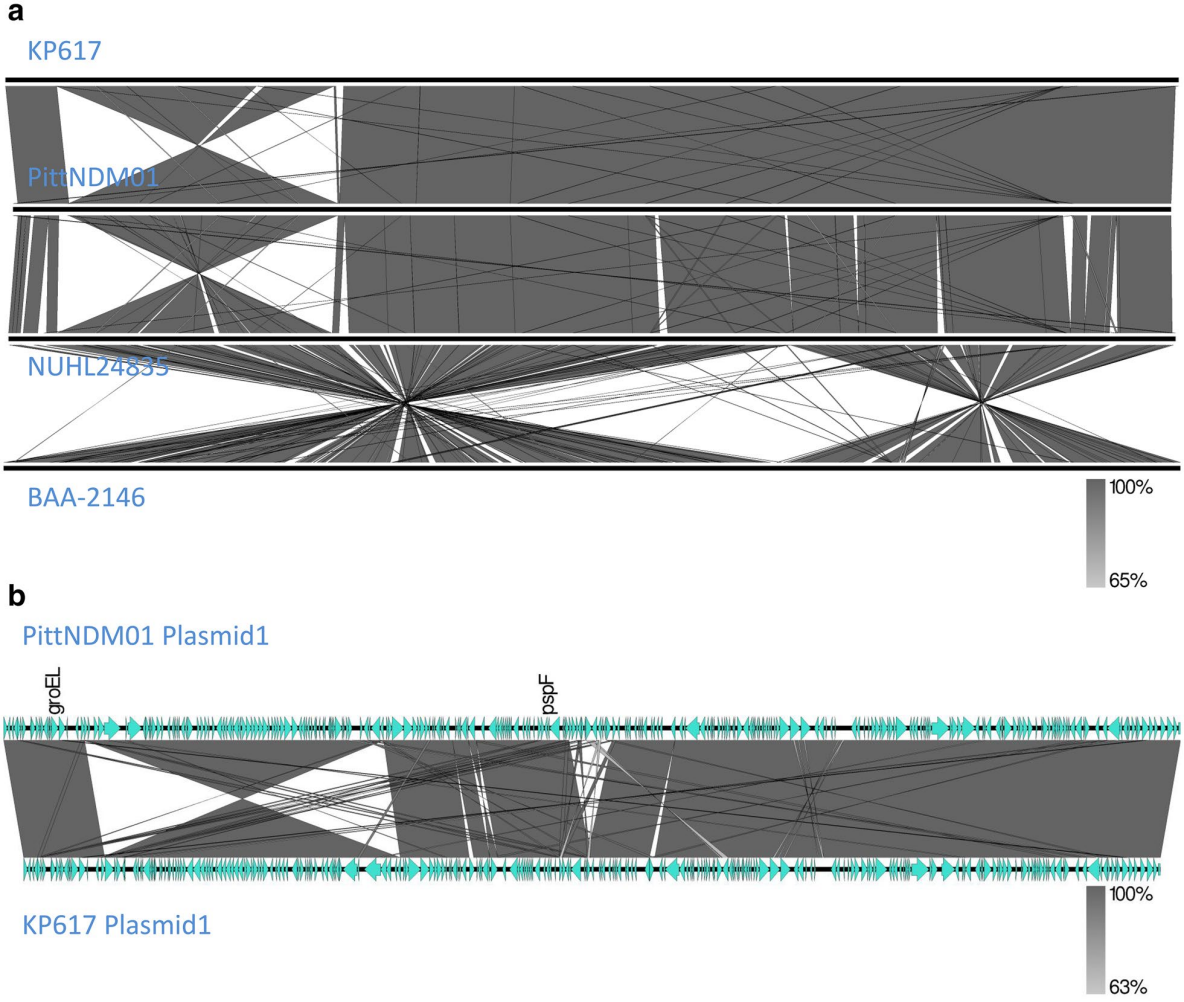
Comparative analysis of genome structures between KP617 and the reference strains PittNDM01, NUHL24835, and ATCC BAA-2146. **a** Comparison of chromosome structure between KP617 and the reference strains. An inversion spanning 233,805 bp to 1,517,597 bp (1 Mb in size) in the KP617 chromosome is shown. **b** Comparison between the structure of plasmid 1 of KP617 and plasmid 4 of PittNDM01. There was a 71 kb inversion, from 18,633 bp to 90,686 bp, in plasmid 1 of the KP617 strain

The comparison of genomic structures of the chromosome indicated the presence of highly conserved structures in the KP617, NUHL24835, and PittNDM01 strains (Fig. 4a). Interestingly, a 1-Mb region (233,805–1,517,597) of the KP617 chromosome was inverted relative to its arrangement in the chromosome of PittNDM01 (1,500,972–225,619). Despite this inversion, KP617 and PittNDM01 exhibited a lower substitution rate (score 20) than NUHL24835 (score 30) (Fig. 3). However, the chromosomal structure of the ATCC BAA-2146 strain, which consisted of two large inverted regions, was significantly different from that of the other strains. In addition, a 71 Kb inversion was found in the sequence of plasmid 1 of KP617 (18,633–90,686) relative to plasmid 1 of PittNDM01 (91,507–19,453); however, the two plasmids were highly homologous to each other (Fig. 4b).

### Antimicrobial resistance genes

Nineteen antibiotic resistance genes were identified in the genome of KP617, 39 in the genome of PittNDM01, 29 in that of ATCC BAA-2146, and nine in that of the NUHL24385 strain (Table 2). The β-lactam resistance genes in the KP617 genome were *bla*_*OXA*-*1*_ and *bla*_*SHV*-*28*_ in the chromosome, *bla*_*NDM*-*1*_ in plasmid 1, and *bla*_*OXA*-*232*_ in plasmid 2; however, genes conferring resistance to colistin and tetracycline were not found (Table 2). Plasmid 2 of KP617, which includes the OXA-232-encoding gene, consists of a 6141-bp sequence; the sequence of this plasmid was identical to that of plasmid 4 of PittNDM01 (100 % coverage and similarity) and the plasmid of *E. coli* (coverage: 100 %, similarity: 99.9 %). Plasmid 2 of KP617, plasmid 4 of PittNDM01 and *E. coli* Mob gene cluster (GenBank accession: JX423831) [12] carried the OXA-232-encoding gene, and pKF-3 of *K. pneumoniae* carried the OXA-181-encoding gene. However, pKF-3 was identical to plasmid 2 of KP617, except in that the insertion sequence IS*Ecp1* was inserted upstream of OXA-181 and included in the transposon Tn*2013* [12, 34].

**Table 2:** Antimicrobila resistance genes of KP617 and the reference strains

Antibiotics	Resistance gene	% identity	Query/HSP length	Predicted phenotype	Accession number	Position^a^			
						KP617	PittNDM01	BAA-2146	NHUL24385
Aminoglycosides	*aacA4*	100	555/555	Aminoglycoside resistance	KM278199			P3_115183..115737	
	*aac(3)*-*IIa*	99.77	861/861		X51534			P2_41114..41974	
	*aac(3)*-*IId*	99.88	861/861		EU022314		P3_64003..64863		
	*aac(6′)*-*Ib*	100	606/606		M21682		P3_2456..3061	P2_82742..83347	
	*aadA1*	100	789/789		JQ480156		P3_3131..3919		
		99.75	792/798		JQ414041		P3_44412..45203		
	*aadA2*	100	792/792		JQ364967	P1_261911..262702	P1_271654..272445		P1_53050..53841
		100	780/780		X68227			C_2297697..2298476	
	*aph(3′)*-*VIa*	98.46	780/780		X07753	P1_4558..5337	P1_4558..5337		
	*armA*	100	774/774		AY220558	P1_267391..268164	P1_277134..277907		
	*rmtC*	100			AB194779			P3_120100..120945	
	*strA*	99.88	804/804		AF321551		P3_29207..30010		
		100						P2_53242..54045	
	*strB*	99.88	837/837		M96392		P3_30010..30846		
		100						P2_52406..53242	
	*aac(6′)Ib*-*cr*	100	600/600	Fluoroquinolone and aminoglycoside resistance	DQ303918	C_612688..613287	C_1122863..1123462		
							P1_136163..136762	P2_38111..38710	
Beta-lactams	*blaOXA*-*1*	100	831/831	Beta-lactam resistance	J02967	C_613418..614248	C_1121902..1122732		
							P1_136893..137723	P2_38841..39671	
	*blaOXA*-*9*	100	840/840		JF703130		P3_3964..4803		
	*blaOXA*-*232*	100	798/798		JX423831	P2_3878..4675	P4_3878..4675		
	*blaNDM*-*1*	100	813/813		FN396876	P1_7770..8582	P1_7770..8582	P3_122191..123003	
	*blaNDM*-*5*	100	813/813		JN104597				P2_10716..11528
	*blaCTX*-*M*-*15*	100	876/876		DQ302097			C_5407907..5408782	
							P3_68389..69264	P2_47128..48003	P1_47694..48569
	*blaTEM*-*1A*	100	861/861		HM749966		P3_5503..6363		
	*blaTEM*-*1B*	100	861/861		JF910132			P2_50825..51685	
			595/861						P1_49351..49945
	*blaSHV*-*11*	100	861/861		GQ407109		P3_57446..58306	C_2612965..2613825	
		99.88						P2_36311..37171	
	*blaSHV*-*28*	100	861/861		HM751101				C_1087615..1088475
		99.88				C_1078475..1079335	C_656815..657675		
	*blaCMY*-*6*	100	1146/1146		AJ011293			P3_72203..73348	
Fluoroquinolones	*aac(6′)Ib*-*cr*	100	600/600	Fluoroquinolone and aminoglycoside resistance	DQ303918	C_612688..613287	C_1122863..1123462		
							P1_136163..136762	P2_38111..38710	
		99.42	519/519		EF636461		P3_2543..3061	P2_82742..83260	
		99.61						P3_115219..115737	
	*QnrB1*	99.85	682/681	Quinolone resistance	EF682133	P1_130519..131200	P1_130247..130928		
	*QnrB58*	98.68	681/681		JX259319			P2_26062..26742	
	*oqxA*	100	1176/1176		EU370913			C_4169699..4170874	
		99.23				C_4847144..4848319	C_4793024..4794199		C_4849531..4850706
	*oqxB*	98.83	3153/3153		EU370913	C_4843968..4847120	C_4789848..4793000	C_4170898..4174050	
		98.79							C_4846355..4849507
Fosfomycin	*fosA*	97.38	420/420	Fosfomycin resistance	NZ_AFBO01000747	C_2957629..2958048	C_2903507..2903926		C_2946180..2946599
		97.14						C_667959..668378	
MLS—macrolide, lincosamide and streptogramin B	*ere(A)*	95.11	1227/1227	Macrolide resistance	AF099140		P3_45289..46515		
	*mph(A)*	100	906/906		D16251			P1_16503..17408	
	*mph(E)*	99.89	885/885		EU294228	P1_271994..272878	P1_281737..282621		
	*msr(E)*	100	1476/1476	Macrolide, Lincosamide and Streptogramin B resistance	EU294228	P1_270463..271938	P1_280206..281681		
Phenicol	*catB3*	100	442/633	Phenicol resistance	AJ009818		P1_137861..138302	P2_39809..40250	
						C_614386..614827	C_1121323..1121764		
	*cmlA1*	99.13			AB212941		P3_42931..44190		
Rifampicin	*ARR*-*2*	100	453/453	Rifampicin resistance	HQ141279		P3_46791..47243		
	*ARR*-*3*				CP002151			C_2298894..2299820	
Sulphonamides	*sul1*	100	927/927	Sulphonamide resistance	CP002151	P1_263120..264046	P1_272863..273789	P3_116160..117086	
	*sul1*	100	837/837		JN581942		P3_41559..42395		
	*sul2*	100	816/816		GQ421466		P3_28331..29146		
Tetracyclines	*tet(A)*	100	1200/1200	Tetracycline resistance	AJ517790			P1_19168..20367	
Trimethoprim	*dfrA1*	100	474/474	Trimethoprim resistance	X00926	C_3627607..3628080	C_3573485..3573958		
	*dfrA12*	100	498/498		AB571791	P1_261006..261503	P1_270749..271246		P1_52145..52642
	*dfrA14*	99.59	483/483		DQ388123		P1_144525..145007	P2_8272..8754	

KP617: C, CP012753.1; P1, CP012754.1; P2, CP012755.1

PittNDM01: C, CP006798.1; P1, CP006799.1; P2, CP006800.1; P3, CP006801.1; P4, CP006802.1

ATCC BAA-2146: C, CP006659.2; P1 (PCuAs), CP006663.1; P2 (PHg), CP006662.2; P3, CP006660.1; P4, CP006661.1

NUHL24385: C, CP014004.1; P1, CP014005.1; P2, CP014006.1

^a^ *C* chromosome, *P* plasmid

The structure of plasmid 1 (273,628 bp in size) of the KP617 strain was similar to that of plasmid 1 (283,371 bp in size) of PittNDM01. A region of about 40 kb in size within plasmid 1 of the KP617 strain, which included the NDM-1-encoding gene, was composed of various resistance genes such as *aadA2, armA, aac(3″)*-*VI, dfrA12*, *msrE, mphE, sul1* and *qnrB1,* and identical (coverage: 100 %, homology: 100 %) to a 40-kb sequence of plasmid 1 of PittNDM01 (Fig. 4b). Adjacent to the NDM-1-encoding gene, a region of about 70 kb in size was inverted in plasmid 1 of KP617 relative to plasmid 1 of PittNDM01. In addition, the OXA-1-encoding gene was identified in PittNDM01 but not in KP617. Transposases were found in a part of the NDM-1-encoding gene cluster (about 10 kb) in plasmid 1 of KP617. Gram-negative bacteria are known to possess a diverse range of transposases; moreover, the sequence of the NDM-1-encoding gene cluster includes a transposon [35, 36]. The partial, or complete, transfer of NDM-1-harboring plasmids between *K. pneumoniae* and *E. coli*, via conjugation, has been shown to result in the emergence of strains resistant to several antimicrobial agents [11, 32, 36, 37].

Following the initial identification of NDM-1 in a *K. pneumoniae* isolate from a patient who had travelled to India in 2008, most NDM-1-producing *K. pneumoniae* isolates have been recovered from patients associated with India; however, in some cases, these strains have been isolated from patients with no history of travelling abroad, or any association with India [38]. These observations suggest that the transfer of the NDM-1- and OXA-232-harboring plasmids between Gram-negative bacteria has resulted in the spread of carbapenem resistance and emergence of strong carbapenem-resistant strains outside the Indian subcontinent.

### Virulence factors

*Klebsiella pneumoniae*, a significant pathogen of human hosts, causes urinary tract infections, pneumonia, septicemia, and soft tissue infections [1]. The clinical features of *K. pneumoniae* infections depend on the virulence factors expressed by the infecting strain [39]. Therefore, we investigated the virulence factors of the present strain and compared these with those of KP617 and the reference strains. A BLAST search was performed against VFDB to identify 117 virulence factors harbored by the KP617 strain (Table 3). All 117 virulence genes of KP617 were also harbored by the reference strains; KP617 did not possess any unique virulence factors. The PittNDM01 strain was also found to possess no unique virulence factors; however, NUHL24835 and ATCC BAA-2146 possessed 3 and 7 unique virulence factors, respectively. The 117 virulence genes of KP617 were classified into 31 the following categories: Iron uptake (30 genes), Immune evasion (12 genes), Endotoxin (11 genes), Adherence (10 genes), Fimbrial adherence determinants (8 genes), Toxin (7 genes), Antiphagocytosis (6 genes), Regulation (5 genes), Acid resistance (3 genes), Anaerobic respiration (2 genes), Cell surface components (2 genes) and Secretion system (2 genes). Among the 117 virulence genes identified, 8 genes were lipopolysaccharide [40]-related genes and 4 genes were capsular polysaccharide [41]-related.

**Table 3:** Virulence genes of KP617 and the reference strains

Strains	Category	Subcategory	Name
KP617, PittNDM01, NUHL24385 and ATCC BAA-2146	Acid resistance	Urease	*ureA, ureB, ureF, ureG, ureH*
	Adherence	Cell wall associated fibronectin binding protein	*ebh*
	Adherence	CFA/I fimbriae	*ibeB*
	Adherence	Flagella	*fleN, fleR, fleS*
	Adherence	Hsp60	*htpB*
	Adherence	Intercellular adhesin	*icaA, icaR*
	Adherence	Listeria adhesion protein	*lap*
	Adherence	OapA	*oapA*
	Adherence	Omp89	*omp89*
	Adherence	P fimbriae	*papX*
	Adherence	PEB1/CBF1	*pebA*
	Adherence	Phosphoethanolamine modification	*lptA*
	Adherence	Type I fimbriae	*fimB, fimE, fimG*
	Adherence	Type IV pili	*comE/pilQ*
	Adherence	Type IV pili biosynthesis	*pilM, pilW*
	Adherence	Type IV pili twitching motility related proteins	*chpD, chpE*
	Adhesin	Laminin-binding protein	*lmb*
	Adhesin	Streptococcal lipoprotein rotamase A	*slrA*
	Adhesin	Streptococcal plasmin receptor/GAPDH	*plr/gapA*
	Adhesin	Type IV pili	*pilD, pilN, pilR, pilR, pilS, pilT*
	Amino acid and purine metabolism	Glutamine synthesis	*glnA1*
	Amino acid and purine metabolism	Leucine synthesis	*leuD*
	Amino acid and purine metabolism	Lysine synthesis	*lysA*
	Amino acid and purine metabolism	Proline synthesis	*proC*
	Amino acid and purine metabolism	Purine synthesis	*purC*
	Amino acid and purine metabolism	Tryptophan synthesis	*trpD*
	Anaerobic respiration	Nitrate reductase	*narG, narH, narI, narJ*
	Anaerobic respiration	Nitrate/nitrite transporter	*narK2*
	Anti-apoptosis factor	NuoG	*nuoG*
	Antimicrobial activity	Phenazines biosynthesis	*phzE1, phzF1, phzG1phzS*
	Antiphagocytosis	Alginate regulation	*algQ, algR, algU, algW, algZ, mucB, mucC, mucD, mucP*
	Antiphagocytosis	Capsular polysaccharide	*cpsB, wbfT, wbfV/wcvB, wbjD/wecB, wza, wzc*
	Antiphagocytosis	Capsule	*cpsF*
	Antiphagocytosis	Capsule I	*gmhA, wcbN, wcbP, wcbR, wcbT, wzt2*
	Cell surface components	GPL locus	*fadE5, fmt, rmlB*
	Cell surface components	MymA operon	*adhD, fadD13, sadH, tgs4*
	Cell surface components	PDIM (phthiocerol dimycocerosate) and PGL (phenolic glycolipid) biosynthesis and transport	*ddrA, mas, ppsC, ppsE*
	Cell surface components	Potassium/proton antiporter	*kefB*
	Cell surface components	Proximal cyclopropane synthase of alpha mycolates	*pcaA*
	Cell surface components	Trehalose-recycling ABC transporter	*lpqY, sugA, sugB, sugC*
	Chemotaxis and motility	Flagella	*flrA, flrB*
	Efflux pump	FarAB	*farA, farB*
	Efflux pump	MtrCDE	*mtrC, mtrD*
	Endotoxin	LOS	*gmhA/lpcA, kdtA, kpsF, lgtF, licA, lpxH, msbA, opsX/rfaC, orfM, rfaD, rfaE, rfaF, wecA, yhbX*
	Endotoxin	LPS	*bplA, bplC, bplF, wbmE, wbmI*
	Endotoxin	LPS-modifying enzyme	*pagP*
	Exoenzyme	Cysteine protease	*sspB*
	Exoenzyme	Streptococcal enolase	*eno*
	Fimbrial adherence determinants	Agf/Csg	*csgD*
	Fimbrial adherence determinants	Fim	*fimA, fimC, fimD, fimF, fimH, fimI*
	Fimbrial adherence determinants	Lpf	*lpfB, lpfC*
	Fimbrial adherence determinants	Stg	*stgA*
	Fimbrial adherence determinants	Sth	*sthA, sthB, sthC, sthD, sthE*
	Fimbrial adherence determinants	Sti	*stiB*
	Glycosylation system	N-linked protein glycosylation	*pglJ*
	Host immune evasion	Exopolysaccharide	*galE, galU, manA, mrsA/glmM, pgi*
	Host immune evasion	LPS glucosylation	*gtrB*
	Host immune evasion	Polyglutamic acid capsule	*capD*
	Immune evasion	LPS	*acpXL, htrB, kdsA, lpxA, lpxB, lpxC, lpxD, lpxK, pgm, wbkC*
	Intracellular survival	LigA	*ligA*
	Intracellular survival	Lipoate protein ligase A1	*lplA1*
	Intracellular survival	Mip	*mip*
	Intracellular survival	Oligopeptide-binding protein	*oppA*
	Intracellular survival	Post-translocation chaperone	*prsA2*
	Intracellular survival	Sugar-uptake system	*hpt*
	Invasion	Ail	*ail*
	Invasion	Cell wall hydrolase	*iap/cwhA*
	Iron acquisition	Cytochrome c muturation (ccm) locus	*ccmA, ccmB, ccmC, ccmE, ccmF*
	Iron acquisition	Ferrous iron transport	*feoA, feoB*
	Iron acquisition	Iron acquisition/assimilation locus	*iraB*
	Iron and heme acquisition	Haemophilus iron transport locus	*hitA, hitB, hitC*
	Iron and heme acquisition	Heme biosynthesis	*hemA, hemB, hemC, hemD, hemE, hemG, hemH, hemL, hemM, hemN, hemX, hemY*
	Iron uptake	ABC transporter	*fagD*
	Iron uptake	ABC-type heme transporter	*hmuT, hmuU, hmuV*
	Iron uptake	Achromobactin biosynthesis and transport	*acsB, cbrB, cbrD*
	Iron uptake	Aerobactin transport	*iutA*
	Iron uptake	ciu iron uptake and siderophore biosynthesis system	*ciuD*
	Iron uptake	Enterobactin receptors	*irgA*
	Iron uptake	Enterobactin synthesis	*entE, entF*
	Iron uptake	Enterobactin transport	*fepA, fepB, fepC, fepD, fepG*
	Iron uptake	Heme transport	*shuV*
	Iron uptake	Hemin uptake	*chuS, chuT, chuY*
	Iron uptake	Iron-regulated element	*ireA*
	Iron uptake	Iron/managanease transport	*sitA, sitB, sitC, sitD*
	Iron uptake	Periplasmic binding protein-dependent ABC transport systems	*viuC*
	Iron uptake	Pyochelin	*pchA, pchB, pchR*
	Iron uptake	Pyoverdine	*pvdE, pvdH, pvdJ, pvdM, pvdN, pvdO*
	Iron uptake	Salmochelin synthesis and transport	*iroE, iroN*
	Iron uptake	Vibriobactin biosynthesis	*vibB*
	Iron uptake	Vibriobactin utilization	*viuB*
	Iron uptake	Yersiniabactin siderophore	*ybtA, ybtP*
	Iron uptake systems	Ton system	*exbB, exbD*
	Lipid and fatty acid metabolism	FAS-II	*kasB*
	Lipid and fatty acid metabolism	Isocitrate lyase	*icl*
	Lipid and fatty acid metabolism	Pantothenate synthesis	*panC, panD*
	Lipid and fatty acid metabolism	Phospholipases C	*plcD*
	Macrophage inducible genes	Mig-5	*mig*-*5*
	Magnesium uptake	Mg2+ transport	*mgtB*
	Mammalian cell entry (mce) operons	Mce3	*mce3B*
	Metal exporters	Copper exporter	*ctpV*
	Metal uptake	ABC transporter	*irtB*
	Metal uptake	Exochelin (smegmatis)	*fxbA*
	Metal uptake	Heme uptake	*mmpL11*
	Metal uptake	Magnesium transport	*mgtC*
	Metal uptake	Mycobactin	*fadE14, mbtH, mbtI*
	Motility and export apparatus	Flagella	*flhF, flhG, fliY*
	Nonfimbrial adherence determinants	SinH	*sinH*
	Other adhesion-related proteins	EF-Tu	*tuf*
	Other adhesion-related proteins	PDH-B	*pdhB*
	Others	MsbB2	*msbB2*
	Others	Nuclease	*nuc*
	Others	VirK	*virK*
	Phagosome arresting	Nucleoside diphosphate kinase	*ndk*
	Protease	Trigger factor	*tig/ropA*
	Proteases	Proteasome-associated proteins	*mpa*
	Quorum sensing	Autoinducer-2	*luxS*
	Quorum sensing systems	Acylhomoserine lactone synthase	*hdtS*
	Quorum sensing systems	*N*-(butanoyl)-l-homoserine lactone QS system	*rhlR*
	Regulation	Alternative sigma factor RpoS	*rpoS*
	Regulation	AtxA	*atxA*
	Regulation	BvrRS	*bvrR*
	Regulation	Carbon storage regulator A	*csrA*
	Regulation	DevR/S	*devR/dosR*
	Regulation	GacS/GacA two-component system	*gacA, gacS*
	Regulation	LetA/LetS two component	*letA*
	Regulation	LisR/LisK	*lisK*
	Regulation	MprA/B	*mprA, mprB*
	Regulation	PhoP/R	*phoR*
	Regulation	RegX3	*regX3*
	Regulation	RelA	*relA*
	Regulation	SenX3	*senX3*
	Regulation	Sigma A	*sigA/rpoV*
	Regulation	Two-component system	*bvgA, bvgS*
	Secreted proteins	Antigen 85 complex	*fbpB, fbpC*
	Secretion system	Accessory secretion factor	*secA2*
	Secretion system	Bsa T3SS	*bprC*
	Secretion system	Flagella (cluster I)	*fliZ*
	Secretion system	Mxi-Spa TTSS effectors controlled by MxiE	*ipaH, ipaH2.5*
	Secretion system	*P. aeruginosa* TTSS	*exsA*
	Secretion system	*P. syringae* TTSS	*hrcN*
	Secretion system	*P. syringae* TTSS effectors	*hopAJ2, hopAN1, hopI1*
	Secretion system	TTSS secreted proteins	*bopD*
	Secretion system	Type III secretion system	*bscS*
	Secretion system	Type VII secretion system	*essC*
	Secretion system	VirB/VirD4 type IV secretion system & translocated effector Beps	*bepA*
	Serum resistance	BrkAB system	*brkB*
	Stress adaptation	AhpC	*ahpC*
	Stress adaptation	Catalase-peroxidase	*katG*
	Stress adaptation	Pore-forming protein	*ompA*
	Stress protein	Catalase	*katA*
	Stress protein	Manganese transport system	*mntA, mntB, mntC*
	Stress protein	Recombinational repair protein	*recN*
	Stress protein	SodCI	*sodCI*
	Surface protein anchoring	Lipoprotein diacylglyceryl transferase	*lgt*
	Surface protein anchoring	Lipoprotein-specific signal peptidase II	*lspA*
	Toxin	Beta-hemolysin/cytolysin	*cylG*
	Toxin	Enterotoxin	*entA, entB, entC, entD*
	Toxin	Hydrogen cyanide production	*hcnC*
	Toxin	Phytotoxin phaseolotoxin	*argD, argK, cysC1*
	Toxin	Streptolysin S	*sagA*
	Toxins	Alpha-hemolysin	*hlyA*
	Toxins	Enterotoxin SenB/TieB	*senB*
	Two-component system	PhoPQ	*phoP, phoQ*
	Type I secretion system	ABC transporter for dispersin	*aatC*
KP617, PittNDM01 and NUHL24385	Antiphagocytosis	Capsular polysaccharide	*cpsA*
	Cell surface components	GPL locus	*pks*
	Cell surface components	Mycolic acid trans-cyclopropane synthetase	*cmaA2*
	Endotoxin	LOS	*lgtA*
	Iron uptake	Pyoverdine receptors	*fpvA*
	Iron uptake	Vibriobactin biosynthesis	*vibA*
	Iron uptake	Yersiniabactin siderophore	*irp1, irp2, ybtE, ybtQ, ybtS, ybtT, ybtU, ybtX*
	Secretion system	EPS type II secretion system	*epsG*
	Secretion system	Trw type IV secretion system	*trwE*
	Secretion system	VirB/VirD4 type IV secretion system & translocated effector Beps	*virB11, virB4, virB9*
	Toxin	RTX toxin	*rtxB, rtxD*
KP617 and PittNDM01	Adhesin	Streptococcal collagen-like proteins	*sclB*
	Chemotaxis and motility	Flagella	*flrC*
	Iron uptake	Yersiniabactin siderophore	*fyuA*

KP617 and PittNDM01 were found to possess two virulence factors that were not present in the other two strains: invasion (encoded by *ail*, attachment invasion locus protein) [42] and Iron uptake (encoded by *fyuA*, Yersiniabactin siderophore) [43].

### Phage-associated regions

Prophages contribute to the genetic and phenotypic plasticity of their bacterial hosts [44] and act as vehicles for the transfer of antimicrobial resistance genes [45] or virulence factors [46]. Six phage-associated regions (KC1–KC5) of the KP617 chromosome and one phage-associated region (KP1) in plasmid 1 of the KP617 strain were identified using the PHAST algorithm (Table 4). With regard to the reference strains, six phage-associated regions were identified in the PittNDM01 strain, six in NUHL24835, and 12 in ATCC BAA-2146.

**Table 4:** Phage-associated regions of KP617 and the reference strains

Strain	Chromosome/plasmid	Region	Region_length (Kb)	Completeness	Score	#CDS	Region_position	Possible phage	GC_percentage (%)
ATCC BAA-2146	Chromosome	AC1	23.3	Questionable	75	14	596765–620097	Entero_P4	43.01
	Chromosome	AC2	52	Intact	100	70	1293924–1345940	Cronob_ENT47670	53.06
	Chromosome	AC3	37.5	Intact	150	48	1785522–1823022	Entero_Fels_2	51.11
	Chromosome	AC4	25.7	Incomplete	50	31	2283748–2309524	Entero_mEpX1	52.98
	Chromosome	AC5	45.6	Intact	110	62	2342458–2388075	Salmon_SEN34	51.79
	Chromosome	AC6	7	Incomplete	30	7	3543581–3550658	Shigel_SfIV	48.73
	Chromosome	AC7	45.1	Intact	106	57	3969834–4015015	Salmon_SPN1S	54.61
	Chromosome	AC8	24.7	Intact	150	31	4128565–4153295	Salmon_RE_2010	56.56
	Chromosome	AC9	25.7	Questionable	90	26	4910621–4936374	Salmon_ST64B	52.32
	Plasmid1	AP1-1	16	Questionable	70	13	5385–21439	Staphy_SPbeta_like	57.65
	Plasmid2	AP2-1	46	Intact	130	38	3924–49935	Stx2_converting_1717	51.29
	Plasmid2	AP2-2	18.1	Questionable	70	23	37308–55427	Staphy_SPbeta_like	50.68
	Plasmid2	AP2-3	18.7	Incomplete	30	21	66337–85097	Entero_P1	51.85
KP617	Chromosome	KC1	59.4	Intact	140	78	187337–246765	Salmon_E1	53.99
	Chromosome	KC2	52.2	Intact	150	51	1148902–1201105	Entero_HK140	54.02
	Chromosome	KC3	37.3	Intact	150	39	1524848–1562220	Salmon_SEN4	50.97
	Chromosome	KC4	43.1	Questionable	90	52	4912300–4955407	Escher_HK639	52.40
	Chromosome	KC5	20	Incomplete	30	17	5015118–5035178	Entero_phiP27	51.93
	Plasmid1	KP1-1	20.7	Incomplete	50	25	123005–143753	Escher_Av_05.	0.4718
NUHL24835	Chromosome	NC1	41.6	Intact	140	47	132925–174606	Entero_HK140	50.75
	Chromosome	NC2	12.8	Incomplete	30	14	1481474–1494341	Thermu_phiYS40	58.36
	Chromosome	NC3	34.7	Intact	150	32	1524859–1559640	Entero_c_1	52.15
	Chromosome	NC4	41.9	Intact	150	52	4283813–4325722	Entero_Fels_2	53.26
	Chromosome	NC5	38.7	Intact	150	45	5082826–5121566	Entero_mEp235	50.24
	Plasmid1	NP1-1	21.4	Incomplete	30	6	65638–87083	Entero_P1	49.29
PittNDM01	Chromosome	PC1	50.8	Intact	130	63	209103–259953	Vibrio_pYD38_A	53.35
	Chromosome	PC2	49.9	Intact	120	65	4847596–4897574	Salmon_SPN3UB	51.59
	Chromosome	PC3	20	Incomplete	30	19	4961006–4981067	Entero_P4	51.92
	Plasmid1	PP1-1	30.8	Questionable	70	22	124082–154939	Vibrio_pYD38_A	48.18
	Plasmid2	PP2-1	34.3	Questionable	70	27	556–34952	Entero_P1	52.30
	Plasmid3	PP3-1	50.3	Intact	150	56	8885–59236	Entero_P1	53.90

Three of the six phages, KC1, KC2 and KC3, in the KP617 strain were intact, whereas the remaining prophages were incomplete (KC5 and KP1) or questionable (KC4) and had a low PHAST score of below 90. Based on the sequence similarity of their genomes, KP617 and PittNDM01 were found to have high similarity to each other (Figs. 2, 3a, b). Concordantly, the profile of prophage DNA in their genomes, as determined via a BLAST search, was similar, and the two strains shared four of the six prophages, whereas two phage regions, KC2 (Entero_HK140) and KC3 (Salmon_SEN4), were specific to the KP617 genome. Furthermore, it was found that one phage-associated region of KP617, namely KC2 (Entero_HK140), exhibited a high similarity to the phage-associated region of the NUHL24835 strain, NC1, with 60 % query coverage and 99 % identity. It should be noted that the strains compared in the present study, i.e. KP617 and the reference strain, ATCC BAA-2146, had no prophages in common.

Investigation of the antimicrobial resistance genes harbored by the strains, which was performed using ResFinder, and comparison with the prophage-associated region, as predicted using PHAST, did not reveal the presence of a prophage-delivered beta-lactamase-encoding gene in the KP617 genome, indicating that prophages did not act as a vehicle for the transfer of antimicrobial resistance genes in this strain. This finding is consistent with previous observations that beta-lactamase-encoding genes are borne by transposons [35, 36]. Bacteriophages are applicable to phage therapy. In particular, bacteriophages have been used as a potential therapeutic agent to treat patients infected with multidrug resistant bacteria [47] and have been used for serological typing for diagnostic and epidemiological typing in *K. pneumoniae* [48]. However, because we did not characterize the phages in KP617, we are not sure whether or not they are active.

## Future directions

*Klebsiella pneumoniae* subsp. *pneumoniae* KP617, which is strongly pathogenic, is known to cause severe nosocomial infections. This strain, as well as the PittNDM01 and NUHL24835 strains in the WGLW2 group, belongs to the sequence type ST14. In this study, we investigated specific antimicrobial resistance genes, virulence factors, and prophages related to pathogenicity and drug resistance in *K. pneumoniae* subsp. *pneumoniae* KP617 via a comparative analysis of the genome of this strain and those of PittNDM01, NUHL24835, and ATCC BAA-2146. Significant homology was observed in terms of the genomic structure, gene content, antimicrobial resistance genes and virulence factors between KP617 and the reference strains; phylogenetic analysis indicated that KP617 is next to PittNDM01, despite the presence of large inversions. Moreover, KP617 shares 98.3 % of its genes with PittNDM01. Despite the similarity in genome sequences and content, there were differences in phage-related genes, plasmids, and plasmid-harbored antimicrobial resistance genes. PittNDM01 harbors two more plasmids and 21 more antimicrobial resistance genes than KP617. In order to elucidate the precise role of these factors in the pathogenicity of KP617, further studies are required.

## Availability of supporting data

*Nucleotide sequence accession numbers* The complete genome sequence of *K. pneumoniae* KP617 has been deposited in DDBJ/EMBL/GenBank under the accession numbers CP012753, CP012754, and CP012755 [49].

## Additional file

10.1186/s13099-016-0117-1 Annotated genes of KP617 and comparison of their sequences with those of the reference strains by using the RAST server.

## Abbreviations

BSR: BLAST score ratio
CDS: coding DNA sequences
HGT: horizontal gene transfer
MLST: multi-locus sequence typing
NDM-1: New Delhi metallo-β-lactamase 1
RAST: Rapid Annotation using Subsystem Technology
ST: sequence type
str: strain
substr: substrain
